# Amorphous–Crystalline Calcium Phosphate Coating Promotes In Vitro Growth of Tumor-Derived Jurkat T Cells Activated by Anti-CD2/CD3/CD28 Antibodies

**DOI:** 10.3390/ma14133693

**Published:** 2021-07-01

**Authors:** Yurii P. Sharkeev, Ekaterina G. Komarova, Valentina V. Chebodaeva, Mariya B. Sedelnikova, Aleksandr M. Zakharenko, Kirill S. Golokhvast, Larisa S. Litvinova, Olga G. Khaziakhmatova, Vladimir V. Malashchenko, Kristina A. Yurova, Natalia D. Gazatova, Ivan G. Kozlov, Marina Y. Khlusova, Konstantin V. Zaitsev, Igor A. Khlusov

**Affiliations:** 1 Laboratory of Physics of Nanostructured Biocomposites, Institute of Strength Physics and Materials Science, Siberian Branch of Russian Academy of Sciences, 634055 Tomsk, Russia; sharkeev@ispms.tsc.ru (Y.P.S.); katerina@ispms.ru (E.G.K.); vtina5@mail.ru (V.V.C.); smasha5@yandex.ru (M.B.S.); 2 Research School of High-Energy Physics, National Research Tomsk Polytechnic University, 634050 Tomsk, Russia; 3 School of Engineering, Far Eastern Federal University, 690090 Vladivostok, Russia; rarf@yandex.ru (A.M.Z.); golokhvast.ks@dvfu.ru (K.S.G.); 4 Center for Immunology and Cellular Biotechnology, Immanuel Kant Baltic Federal University, 236029 Kaliningrad, Russia; hazik36@mail.ru (O.G.K.); vlmalashchenko@kantiana.ru (V.V.M.); kristina_kofanova@mail.ru (K.A.Y.); n_gazatova@mail.ru (N.D.G.); 5 Department of Organization and Management in the Sphere of Circulation of Medicines, Institute of Postgraduate Education, I.M. Sechenov Federal State Autonomous Educational University of Higher Education—First Moscow State Medical University of the Ministry of Health of the Russian Federation (Sechenov University), 119991 Moscow, Russia; immunopharmacology@yandex.ru; 6 Department of Pathophysiology, Siberian State Medical University, 634050 Tomsk, Russia; marikhl@mail.ru; 7 Siberian Federal Scientific and Clinical Center of the Federal Medical-Biological Agency, 636070 Seversk, Russia; zaitsev-kv@mail.ru; 8 Research School of Chemistry and Applied Biomedical Sciences, National Research Tomsk Polytechnic University, 634050 Tomsk, Russia; 9 Department of Morphology and General Pathology, Siberian State Medical University, 634050 Tomsk, Russia

**Keywords:** micro-arc oxidation, amorphous–crystalline structure, microstructure, electrical potential, zeta potential, Jurkat T cell culture, gene expression, cytokine secretion

## Abstract

A modern trend in traumatology, orthopedics, and implantology is the development of materials and coatings with an amorphous–crystalline structure that exhibits excellent biocopatibility. The structure and physico–chemical and biological properties of calcium phosphate (CaP) coatings deposited on Ti plates using the micro-arc oxidation (MAO) method under different voltages (200, 250, and 300 V) were studied. Amorphous, nanocrystalline, and microcrystalline statesof CaHPO_4_ and β-Ca_2_P_2_O_7_ were observed in the coatings using TEM and XRD. The increase in MAO voltage resulted in augmentation of the surface roughness *R*_a_ from 2.5 to 6.5 µm, mass from 10 to 25 mg, thickness from 50 to 105 µm, and Ca/P ratio from 0.3 to 0.6. The electrical potential (EP) of the CaP coatings changed from −456 to −535 mV, while the zeta potential (ZP) decreased from −53 to −40 mV following an increase in the values of the MAO voltage. Numerous correlations of physical and chemical indices of CaP coatings were estimated. A decrease in the ZP magnitudes of CaP coatings deposited at 200–250 V was strongly associated with elevated *hTERT* expression in tumor-derived Jurkat T cells preliminarily activated with anti-CD2/CD3/CD28 antibodies and then contacted in vitro with CaP-coated samples for 14 days. In turn, in vitro survival of CD4^+^ subsets was enhanced, with proinflammatory cytokine secretion of activated Jurkat T cells. Thus, the applied MAO voltage allowed the regulation of the physicochemical properties of amorphous–crystalline CaP-coatings on Ti substrates to a certain extent. This method may be used as a technological mechanism to trigger the behavior of cells through contact with micro-arc CaP coatings. The possible role of negative ZP and Ca^2^^+^ as effectors of the biological effects of amorphous–crystalline CaP coatings is discussed. Micro-arc CaP coatings should be carefully tested to determine their suitability for use in patients with chronic lymphoid malignancies.

## 1. Introduction

Bioceramic materials based on calcium orthophosphates (CaPO_4_) are actively used in biomaterials [1,2]. These calcium phosphate (CaP) biomaterials are the most suitable for supporting processes of osseointegration and treatment of bone defects because their structure and chemical composition are similar to the mineral component of mammalian bones and teeth [3,4]. Compounds such as hydroxyapatite (HA), tricalcium phosphate (TCP), octacalcium phosphate (OCP), and others take an active part in bone healing, bone replacement, and reconstruction of bone defects [5,6]. However, the main disadvantages of bioceramic materials are their unreliable mechanical properties and pronounced fragility [1,2]. This is why metals are widely used as materials for creating structurally reliable orthopedic and dental implants [7].

The surface of implants plays an essential role in the interaction with living tissue [7,8]. In some cases, fibrous tissue forms at the implant interface, which can lead to its loosening and even loss. The modification of metal implants by coating deposition is one of the most suitable and useful methods for improving their surface properties, such as in terms of biocompatibility and biological activity, to intensify osteoinduction and angiogenesis and prevent bacterial growth [8]. 

The latest trend in traumatology, orthopedics, and dental implantology is to develop materials and coatings with amorphocrystalline structures such as bioglass and glass-ceramics, which exhibit excellent biocompatibility, no cytotoxicity, and good mechanical properties [7,8,9,10,11,12]. A unique combination of crystalline and amorphous phases and crystal sizes and their distribution in an amorphous matrix allows regulation of the physicochemical and mechanical properties of materials and coatings [9,10,11]. Glass-ceramic materials have significant capability to form a bone-like hydroxyapatite layer when interacting with body fluid due to R-OH active groups forming on their surface [11,12,13,14]. Metal ions typically present in a ceramic matrix can actively participate in metabolic processes of the human body and influence enzymes to promote the formation of new bone tissue [12,13].

The synthesis of a coating on the metal surface can occur with the help of such methods as electrophoretic deposition [7], pulsed laser deposition [8,12], sol–gel coatings [14], high-frequency magnetron sputtering [15], and other methods. The plasma electrolytic oxidation (PEO) or micro-arc oxidation (MAO) method is a flourishing, promising, and simple method for surface treatment that allows generating ceramic-like coatings [13,16,17,18,19]. This method makes it possible to obtain rough, porous coatings on implants with complex shapes [20,21].

The formation of the coating in a micro-arc discharge is associated with the occurrence of high-temperature processes in the zone of local micro-arc discharges under the influence of an external high-voltage source [16]. The process of coating deposition occurs due to oxidation of the metal substrate as well as due to the interaction of electrolyte components with a metal substrate and with each other. As a result of high-temperature plasma-chemical reactions, a ceramic-like layer is formed on the metal surface [18,19]. Due to the high intensity and nonstationarity of micro-arc processes, the ceramic-like layer may contain both an amorphous and crystalline phase. Previous studies have shown [20,21,22] that ceramic coatings deposited by the MAO method in electrolytes containing HA or β-TCP powder included amorphous calcium phosphates and crystalline phases such as dicalcium phosphate anhydrous (DCPA, monetite), calcium pyrophosphate (CPP), and HA and TCP in αor β forms. The amorphous calcium phosphates and monetite and α-TCP can actively dissolve in the biological environment, stimulating biomineralization and the formation of the HA layer [4,23,24].

Da Rocha et al. [25] demonstrated that when hDPSCs contacted with monetite coating containing 5 mol% Sr, they exhibited the highest viability and proliferative activity compared to the coatings with dicalcium phosphate dihydrous (DCPD, brushite) and HA. On the other hand, the authors [26] describe hydroxyapatite and brushite coatings that have high mechanical properties and corrosion resistance in combination with bioactivity. Sasikumar et al. [27] reported biocompatible brushite coatings obtained on the surface of magnesium alloys. DCPD coatings demonstrated super hydrophilic properties and high biocompatibility as well as excellent corrosion resistance. 

The surface properties of CaP coatings such as chemical composition, topography, zeta potential, and hydrophilicity are significant since they determine the osteogenic properties of the coatings [28,29,30,31,32]. Many studies have been conducted in recent years to identify the reasons for bone reparation caused by biomaterials [33]. However, there is still no clear understanding of the biological mechanisms involved in the bone formation process stimulated by biomaterials. Osteoimmunology is a rapidly developing direction of scientific research [34]. In this context, the immune cells are described as regulators of inflammation and bone tissue regeneration switching [35] mediated, in part, by CaP materials [36,37]. 

CaPs are among the best grafts to repair and substitute damaged bone [38]. However, there is still no clear evidence of which pathways are involved in the process of bone regeneration caused by biomaterials in tumor patients. Little is known about the interactions of CaP materials with leukemic immune cells. We have recently demonstrated in vitro downregulation of morphofunctional reaction of human Jurkat T lymphoblast-like leukemia-derived cell line (Jurkat T cells) and the possible role of negative surface charge of micro-arc CaP coating on titanium (Ti) implants for the development of tumor cell hypoergy [39]. Jurkat T cells change their electrostatic charge when the cells are activated with microbeads coated with anti-CD3/CD28 antibodies [40]. In turn, cell activation triggers a hyperergy of normal T lymphocytes [41] and leukemic Jurkat T cells to enhance their motility and tissue infiltration [42].

Therefore, the aim of this investigation is to study the physico–chemical properties (microstructure; phase composition; roughness, thickness, and mass; electrostatic and zeta potentials) of CaP coatings on Ti plates prepared by MAO method at the different voltages of 200–300 V in relation to the in vitro examination of morphofunctional behavior of tumor Jurkat T cells preliminarily activated with anti-CD2/CD3/CD28 antibodiesand contacted with test CaP-coated samples.

## 2. Materials and Methods

### 2.1. Pretreatment and MAO Treatment Procedures

Commercial pure titanium (ASTM grade 2) plates (thickness, 1 mm; width, 10 mm, length, 10 mm) (VSMPO-AVISMA Corp., Verkhnaya Salda, Russia) were used in this study. The sample pretreatment procedures include polishing on silicon carbide paper with a grade ranging to P2000, ultrasonic cleaning (Elmasonic S, Elma Schmidbauer GmbH, Singen, Germany) in distilled water and ethanol for 10 min, and air-drying.

The Microarc-3.0 installation (ISPMS SB RAS, Tomsk, Russia) was used to carry out the MAO process and consists of a pulsed DC power supply, titanium electrolytic bath as an electrode (cathode), sample holder as the counterelectrode (anode), and PC for process control. In order to synthesize the CaP coatings, the electrolyte contained 5 wt.% nanosized HA (Ca_10_(PO_4_)_6_(OH)_2_), 7 wt.% CaCO_3_, 27 wt.% H_3_PO_4_, and distilled water as a balance [43,44]. Stoichiometric HA powder consisting of aggregates of round-shaped nanocrystallites with a size less than 50 nm was produced by solid-phase mechanochemical synthesis [45]. An acidic suspension electrolyte (pH = 1–2) incorporating an undissolved nano-dispersed HA was obtained. The MAO process was carried out in an anodic potentiostatic regime with following parameters: pulse frequency of 50 Hz; pulse duration of 100 µs; time of 10 min; and varied applied voltage of 200, 250, and 300 V [39,43,44].

### 2.2. CaP Coating Characterization

Coating morphology and thickness were analyzed by scanning electron microscopy (SEM, LEO EVO 50, Carl Zeiss, Oberkochen, Stuttgart, Germany). The thickness was measured using SEM images of the coating cross-sections according to the ASTM E1382-9 standard protocol. Elemental composition was examined using electron dispersive X-ray spectroscopy (EDX) on an INCA system (Oxford Instruments, High Wycombe, UK) coupled to SEM. Coating microstructure was observed by transmission electron microscopy (TEM, JEM 2100, JEOL, Tokyo, Japan), operating at 200 kV. A replica with the particles removed from the CaP coating layer was studied by TEM. The SEM, TEM, and EDX studies were conducted in the “Nanotech” Common Center for Collective Use (ISPMS SB RAS, Tomsk, Russia).

Phase composition was identified by X-ray diffraction (XRD) in Bragg–Brentano geometry using Shimadzu XRD-6000 equipment. XRD measurements were collected with Cu Kα radiation (λ = 1.54 Å), operating at 40 kV and 30 mA in a 2*θ* range between 10° and 80°, with a copper monochromator coupled and scan speed of 2.0°/min. For the crystalline phase indentation, the International Centre for Diffraction Data (ICDD) database was used as a reference. The equipment was provided by the Tomsk Materials Science Center for Collective Use (National Research Tomsk State University, Tomsk, Russia).

The integral electrical potential (EP) of the coatings was measured by the Eguchi method (the method of lifting the electrode) [46] in the air at ambient conditions as in the previous work [47]. 

The mass of the bare and coated samples was measured using a digital micro-analytical balance (GR-202, A&D Company, Tokyo, Japan). Surface roughness indices were measured by a contact profilometer (Hommel-Etamic T1000 Basic, Jenoptik, Jena, Germany) according to [48]. The traverse length and rate of the measured profile were 6 mm and 0.5 mm/s, respectively. Previously [20,47], it was shown that the definition of the average roughness index *R_a_* is sufficient to describe the surface roughness since it correlates directly proportionally with other roughness parameters (*R_z_*, *R_max_*, etc.).

The surface zeta potential (ZP) was determined with a SurPASS 3 electrokinetic surface analyzer (Anton Paar GmbH, Graz, Austria) by measuring the streaming current and the streaming potential in the Adjustable Gap Cell. In this experiment, two CaP coated samples (thickness, 1 mm; width, 10 mm; length, 20 mm) were attached to the Adjustable Gap Cell strictly parallel at a distance of 100 μm from each other. Such conditions are required to measure the capillary streaming potential in the electrolyte. The 0.001 mol/L KCl with pH~7 was used as the base electrolyte. The electrolyte pH varied from 5.4 to 8.0 by adding different amounts of 0.05 mol/L NaOH to the base electrolyte.

### 2.3. Jurkat T Cell Culture

The Cell Bank of the Institute of Cytology (Institute of Cytology, Russian Academy of Sciences, Saint Petersburg, Russia) provided the Jurkat T-cell culture. The medium is based on αMEM (Sigma-Aldrich, St. Louis, MO, USA), supplemented with 10% inactivated (for 30 min at 56 °C) fetal bovine serum (Sigma-Aldrich, St. Louis, MO, USA), 100 U/mL penicillin/streptomycin (Sigma-Aldrich, St. Louis, MO, USA), and 0.3 mg/mL L-glutamine (Sigma-Aldrich, St. Louis, MO, USA). Cell viability was assessed by flow cytometry (FC) using annexin V-fluorescein isothiocyanate (FITC) (Abcam, Cambridge, UK) and propidium iodide (PI, Sigma Aldrich, St. Louis, MO, USA) with the MACS Quant FL7 system (Miltenyi Biotec, Bergisch Gladbach, Germany) [36]. The initial viability of the cells was 95%. αMEM was used, instead of RPMI-1640, as medium because the leukemic cell culture had insufficient secretory activity in RPMI-1640 over 14 days (Table S1). 

To produce cell activation, a 10 µL aliquot containing 2 × 10^6^ of the anti-biotin MACSiBead^™^ Magnetic T-cell Activation/Expansion/Expansion Kit particles (Miltenyi Biotec, Bergisch Gladbach, Germany) loaded with antibodies to CD2, CD3, and CD28 antigens wad added once to the cell suspension. Anti-biotin MACSiBead^™^ particles mimicked the stimulatory signal produced by antigen-presenting cells (APCs) and served as an activator for dormant T cells. The ratio between Jurkat T cells and the activating particles was 1:2.

Ti substrate coated with CaP was added to each well of a 12-well flat-bottom plate (Orange Scientific, Braine-l’Alleud, Belgium). When culturing the cells for 14 days, three CaP-coated samples were used. For each group, samples were prepared at different applied voltages. The test samples were not added to the control group. The cells were cultured for 14 days at an air humidity of 95% and 5% CO_2_ at 37 °C. The medium in Jurkat T cells culture was replaced every 3–4 days.

After culturing, cells were harvested to measure membrane antigen presentation, apoptosis, necrosis, and hTERT gene expression. Spontaneous secretion induced by CaP coating was measured in cell culture supernatants as described earlier [39].

The Local Ethics Committee of Innovation Park, Immanuel Kant Baltic Federal University, Kaliningrad, Russia approved manipulations with human cells in vitro (Permission No. 2 from 6 March 2017).

### 2.4. Jurkat T Cell Viability and Immunophenotype Detection

After 14 days of cultivation, the viability of Jurkat T cells in vitro was assessed using a MACS Quant flow cytometer (Miltenyi Biotec, Bergisch Gladbach, Germany). The cells were incubated for 10 min in 195 μL binding buffer and 5 μL annexin V-FITC (Abcam, Cambridge, MA, USA). Cells were washed and resuspended in 190 μL binding buffer and 10 μL PI solution (Abcam, Cambridge, MA, USA). The resulting mixture was analyzed by FC. The percentage of living and dead (necrotic or apoptotic) cells was measured according to the manufacturer’s protocol. Cell immunophenotype analysis was performed using monoclonal antibodies (MAb) according to the manufacturer’s instructions.

MAb was labeled with violet blue (VioBlue), fluorescein (FITC), phycoerythrin (PE), allophycocyanin (APC), or phycoerythrin cyanine 7 (PE-Cy7), as described elsewhere [39]. Before staining, Jurkat T cells were washed with phosphate-buffered saline (pH = 7.2). Cells were incubated with a cocktail of monoclonal antibodies against CD45, CD3, CD8, CD4, and CD25 (Abcam, Cambridge, UK) and CD45RA, CD45RO, CD95, and CD71 (e-Bioscience, San Diego, CA, USA). The gating strategy and algorithm for cytometry of the CD45^+^ CD3^+^ subpopulation were previously described [49]. 

The analysis of labeled MAb cells was performed using a MACS Quant flow cytometer according to the manufacturer’s protocol (Miltenyi Biotec, Bergisch Gladbach, Germany). Analysis of the FC results was performed using KALUZA software (Beckman Coulter, Brea, CA, USA).

### 2.5. Gene Expression in Jurkat T Cells 

Evaluation of the expression of differentiation genes (*U2AF1L4*, *GFI1*, and *hnRNPLL*) and *hTERT* (human telomerase reverse transcriptase) by Jurkat T cells was assessed after 14 days of cultivation. Total RNA was isolated from samples using the RNA extract kit (Eurogen, Moscow, Russia) following the manufacturer’s instructions. The resulting total RNA (100 ng) was reverse transcribed into cDNA using reverse transcriptase MMLV (Eurogen, Moscow, Russia) oligo(dT) 23 primer (20 μM) (Beagle, Moscow, Russia). For clarity, Table 1 shows the oligonucleotide primers that were used (Beagle, Moscow, Russia).

According to the previously described scheme [50], the analysis of multiplex polymerase chain reaction (PCR) in triplicate was carried out. For this, special TaqMan probes and primers at a concentration of 10 pM (Beagle, Moscow, Russia), as well as qPCRmixHS reagents (Evrogen, Russia), were used. The work was carried out on a CFX96 qPCR instrument (Bio-Rad, Hercules, California, CA, USA). The sizeable ribosomal protein (*RPLPO*) gene was used as the reference gene.

The second derivative maximum method was used to analyze the PCR results. A modified Pfaffl formula for different amplification efficiency was used to calculate the relative expression levels of target genes. The ratio of the expression of the target gene to the reference gene expression was taken as the basis for a relative quantification (relative quantification), which is sufficient to study the physiological levels of gene expression.

### 2.6. Evaluation of Cell Secretion

Flow fluorimetry (FF) was performed to assess the secretome of activated T cells during co-cultivation with the test materials. For this, an automated processing system (Bio-Plex Protein Assay System, Bio-Rad, Hercules, CA, USA) and a cytokine analysis system (Bio-Plex Pro Human Cytokine 27-Plex Panel, Bio-Rad, Hercules, CA, USA) were used. This test system measures the concentration (pg/mL) of human cytokines and chemokines such as tumor necrosis factor alpha (TNFα); basic fibroblast growth factor (FGFb); vascular endothelial growth factor (VEGF); platelet growth factor (PDGF-BB); granulocyte-macrophage colony-stimulating factor (GM-CSF); granulocyte colony-stimulating factor (G-CSF); interleukins: (IL)-1β, IL-1Ra, IL-2, IL-4, IL-5, IL-6, IL-7, IL-8, IL-9, IL-10, IL-12, IL-13, IL-15, IL-17; interferon gamma (IFN-γ); gamma-induced interferon protein 10 (IP-10; chemokine 10 with motif CXC (CXCL10)); chemoattractant protein-1 of monocytes (MCP-1; chemokine (motif CC) ligand 2 (CCL2)); inflammatory macrophage protein 1 alpha (MIP-1α; CCL3); MIP-1β (CCL4); regulated upon activation, and normal T cell expressed and secreted (RANTES; CCL5); and eotaxin [39]. All work was carried out following the manufacturer’s instructions.

### 2.7. Statistical Analysis

Data analysis was carried out using the hypothesis testing methods and descriptive statistics methods performed in the standard STATISTICA 13.3 software package for Windows. The following distribution parameters were calculated in the work: median (Me) and 25% (Q1) and 75% (Q3) quartiles. The data normality was checked using the Kolmogorov–Smirnov test. The nonparametric Mann–Whitney U test was used to assess the statistical significance of differences. The relationship between the studied parameters was analyzed using Spearman’s rank correlations (*rs*) and regression (*r*) analysis. Differences were considered statistically significant at a significance level of *p* < 0.05.

## 3. Results

### 3.1. Characterization of CaP Coatings

**Figure 1:**
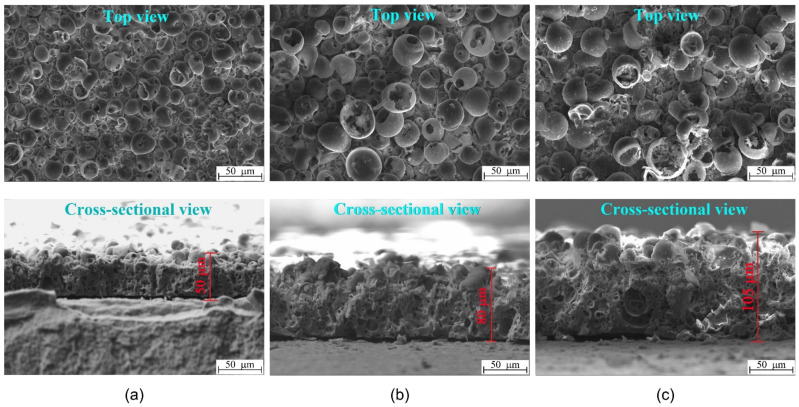
SEM images of the surface and cross-sectional CaP coatings deposited at 200 V (**a**), 250 V (**b**), and 300 V (**c**).

SEM images represent the surface and cross-sectional morphology of the MAO CaP coatings deposited at the applied voltages of 200, 250, and 300 V (Figure 1). As can be seen, the coatings are characterized by a complex hierarchical structure: numerous adjacent pores and pore channels represent the internal morphology, and on the surface, there are sphere-shaped structural elements (spheres) with internal pores and pores located in the spaces between the spheres.

When the MAO voltage was increased from 200 to 300 V, the following morphological transformations in the coatings were observed (Figure 1): the thickness increased from 50 to 105 μm; the spheres and pores increased in sizes, and the spheres were partially destroyed; the plate-like crystals (up to 15 μm in length) were formed inside the destroyed hemispheres on the coating surface; local macropores of 15–30 μm in size were formed near the substrate inside the coatings. These morphological transformations in the coatings inevitably led to an increase in average roughness (*R*_a_) from 2.5 to 6.5 µm, in mass from 10 to 25 mg, and in the sizes of the structural elements (the average size of spheres was from 11.0 to 25.3 µm; the average size of pores was from 2.4 to 7.0 µm). The linear dependences of these coating characteristics on the applied stress value were performed in our previous work [39].

**Figure 2:**
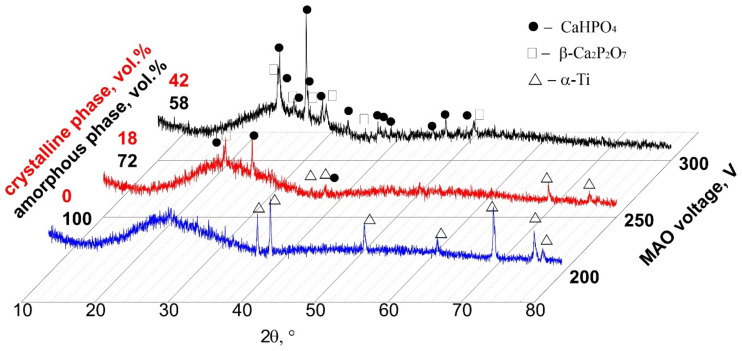
XRD patterns of the MAO CaP coatings formed at 200, 250, and 300 V.

XRD studies showed that the CaP coatings formed at voltages of 200–250 V were mainly in the X-ray amorphous state (Figure 2). The obvious region of diffuse scattering from the quasi-amorphous phase in the range of 2*θ* = 20–38° and weak reflections from a single Ti phase (ICDD #44-1294) of the substrate were observed in the corresponding XRD patterns. On the other hand, XRD patterns of the coatings formed at higher voltages of 250–300 V included the region of diffuse scattering and reflections from the crystalline phases of CaHPO_4_ (monetite; ICDD #09-0080) and β-Ca_2_P_2_O_7_ (ICDD #09-0346). With an increase in the applied voltage from 250 to 300 V, the number of reflections from CaHPO_4_ and β-Ca_2_P_2_O_7_ phases and their intensity increased significantly. As a result, the volume of the full crystalline phase in the coatings increased from 0 to 42 vol.%, while the volume of the amorphous phase decreased from 100 to 58 vol.%. Thus, an increase in the MAO voltage led to the coating structure transforming from the amorphous phase into the amorphous and crystalline states. These XRD data are in good agreement with the SEM results, indicating the plate-shaped monetite crystals on the surface of coating prepared at 300 V (Figure 1c).

**Figure 3:**
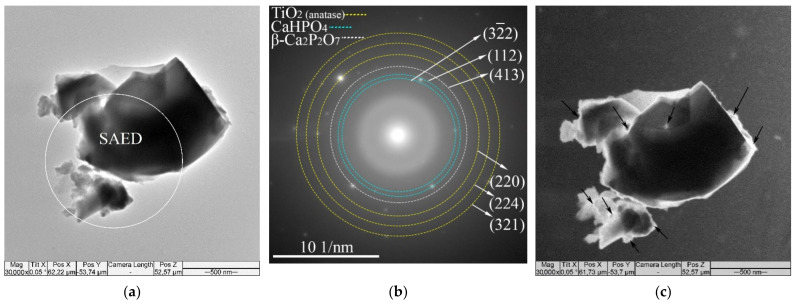
BF TEM (**a**) and DF TEM (**c**) images and SAED pattern (**b**) of the particles of the MAO CaPcoatings. The area where SAED was conducted is marked with a white circle (**a**). Nanocrystallites of the CaHPO_4_ phase in the reflex (112) are marked with black arrows (**c**).

The TEM studies revealed that the MAO CaP coatings deposited at different voltages had an amorphous–nanocrystalline microstructure. Bright field (BF) and dark field (DF) TEM images with selected area electron diffraction (SAED) patterns for fragments of the MAO coatings are shown in Figure 3. Both diffused halos from the amorphous phase and weak point reflexes from crystalline phases were observed in SAED patterns (Figure 3b). The interpretation of the SAED patterns revealed the same crystalline phases that were observed in XRD patterns (Figure 2): β-Ca_2_P_2_O_7_ (ICDD #09-0346), CaHPO_4_ (ICDD #09-0080) and TiO_2_ anatase (ICDD #21-1272). The nanosized crystallites of the CaHPO_4_ phase in the reflex (112) along the boundaries of the coating particles are observed in the DF TEM images (Figure 3c). Reflexes of TiO_2_ anatase observed on SAED (Figure 3b) correspond to the oxide intermediate sublayer in the interface with the Ti substrate.

**Figure 4:**
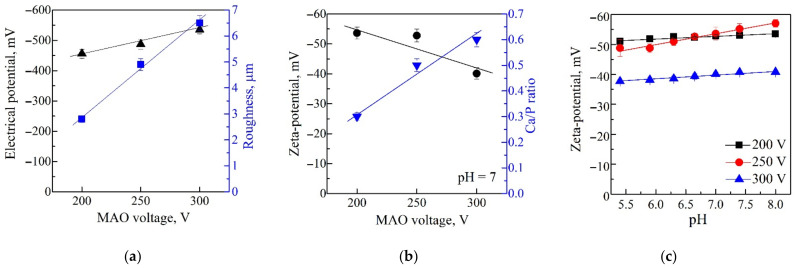
Graphs of the coating surface EP and surface roughness *R_a_* against the applied voltage (**a**); the coating surface ZP and atomic Ca/P ratio against the applied voltage (**b**); the coating surface ZP against the electrolyte pH (**c**).

The Eguchi method showed that all the CaP coatings had negatively charged surfaces with EP values in the range of −456 to −535 mV, depending on the value of the applied MAO voltage (Figure 4a). A close linear dependence (r = 0.93; *p* < 0.0003; *y* = −302 − 0.77*x*) of EP magnitude on applied voltage was estimated (not shown). As shown in Figure 1a, the EP negative values increased with increasing MAO voltages. This effect can be due to the increase in the coating surface roughness *R_a_* from 2.5 to 6.5 µm, and, consequently, the increase in the free surface area. As a result, there was an increase in the number of negatively charged hydroxyl and phosphate groups on the coating surface.

In this study, the surface ZP of the coatings and the EP have negative values. However, these negative values were significantly lower than the EP values since they varied in the range of −40 to −53mV (Figure 4b). This result is the opposite of the EP data (Figure 4a,b). This may be attributed to the different mechanisms of EP and ZP formation. The studied CaP coatings ZP varied in the range from −57 to −38 mV with an increase in pH from 5.4 to 8 units. All test samples had a negative charge at all pH values (Figure 4c). It can be seen that the surface relief had a more significant effect on the electrostatic potential value (Figure 4a), and the zeta potential was more influenced by the chemical composition of the coating (Figure 4b). Significant neutralization of the surface negative ZP may result from the deposition of potassium cations and hydrogen protons from the KCl electrolyte to the sample.

The coating formed at a low voltage of 200 V had a low Ca/P ratio of 0.3 and a ZP value of −53 mV. The increase in the MAO voltage from 250 to 300 V led to powerful discharges. The Ca^2+^ ions were involved in the process more intensively; thus, CaHPO_4_ and β-Ca_2_P_2_O_7_ crystalline phases were formed in the coatings (Figure 2); therefore, the Ca/P ratio increased up to 0.6 (Figure 4b). As a result, the ZP value shifted to a less negative region. It became −40mV, partly due to charge compensation by positive charged coatings areas, perhaps because of Ca^2+^ precipitation from the solution. Thus, the coated sample situated in an aqueous solution for ZP measurement did not lead to full compensation of the coating charge. However, the ZP magnitude was statistically reduced on surfaces prepared at an MAO voltage of 300 V in comparison to those prepared with 200–250 V (Figure 4b).

Therefore, CaP coatings deposited at 300 V were distinct from all others, and an MAO voltage of 200–250 V was used to prepare test samples for biological experiments.

### 3.2. In Vitro Cytological Properties of CaP Coatings

#### 3.2.1. Viability and Immunophenotype of Activated Jurkat T Cells

Preactivation of Jurkat T cells with anti-CD2/CD3/CD28 antibodies that mimic the stimulating signal produced by APCs led to a statistically increased viability of tumor-derived cells after 14 days of culture (Table S2). The rough CaP-coated Ti samples enhanced cell survival through significantly reducing the numbers of apoptotic and necrotic cells compared with the control culture (Table S2).

The majority of viable CD45^+^CD3^+^ Jurkat T cells in the control culture expressed the CD4^+^ (62%) and CD71^+^ (70%)profile of CD45RA^+^ naïve cells (not activated by the antigen, 99%), with subsets of CD4^+^ T helpers expressing markers of early (approximately 28% of the CD25 subset) and late (39% of CD95^+^ cells) activation and apoptosis (Table S2). Cell stimulation with anti-CD2/CD3/CD28 antibodies strongly augmented (*p* < 0.05) the number of CD4^+^ cells (from 62.16% to 84.8%) and their CD71^+^ and CD95^+^ subsets (by 7.52% and 32.32%, respectively). Thereby, the portion of CD4CD45RA^+^ naïve cells statistically decreased, with the control. CaP-coating influence on an immunophenotype of activated tumor cells was of little value, excluding a 1.8-fold drop in CD8^+^ cell proportion compared with the control culture (Table S2). 

Thus, CaP coating on the Ti sample promoted the in vitro survival of CD4^+^ subsets of tumor-derived Jurkat T cells activated by anti-CD2/CD3/CD28 antibodies.

#### 3.2.2. Secretion Capacity of Activated Jurkat T Cells

The spontaneous secretory activity in a 14-day control culture of naïve Jurkat T cells was low (concentrations of 26 molecules less than 100 pg/mL, Table S3); simultaneously, the VEGF median concentration (1735 pg/mL) was extremely high (more than 1 ng/mL) according to [51].

Anti-CD2/CD3/CD28 antibodies modified the profile of biomolecules secreted by Jurkat T cells. On the one hand, a 1.6–19-fold decrease in the concentrations of proinflammatory cytokines (IL-6), growth factors (G-CSF), and chemokines (IL-8, eotaxin, MCP-1) was observed. On the other hand, the levels of VEGF, IL-12, and MIP-1β increased 1.2–1.4 times after 14 days of culture (Table S3).

The secretory spectrum of activated Jurkat T cells in the presence of CaP-coated Ti substrates was similar to the cell culture without test samples. However, the secretion of proinflammatory chemokines MIP-1α and MIP-1β was statistically higher than that in both the control and activated cultures of tumor cells (Table S3). A close direct correlation (*r_S_* = 0.81, *p* < 0.001, *n* = 12) between the *R_a_* index of CaP surface and an increased MIP-1β concentration was noted.

Thus, CaP coating on the Ti substrate maintained the proinflammatory capacity of activated Jurkat T cells in the 14-day culture. 

#### 3.2.3. hTERTExpression in Activated Jurkat T Cells Contacted with CaP-Coated Ti Substrates

Tumor-derived Jurkat T cells preactivated with anti-CD2/CD3/CD28 antibodies and contacted with the micro-arc CaP coating for 14 days showed a 4.5-fold upregulation in *hTERT* expression compared with activated malignant cells cultured without test samples. 

A decrease in the ZP magnitudes (Figure 4b) of negatively charged CaP coatings deposited at 200–250 V was accompanied by elevated *hTERT* expression in Jurkat T cells (r = 0.73; *p* < 0.03). Hence, the influence of ZP on transcription of the hTERTgene, which regulates Jurkat T cell proliferation and survival [52], cannot be excluded. 

The physical features of test samples (*R_a_* index, thickness, and mass) closely correlated (*r_S_* > 0.95; *p* < 0.001) with GFI1 gene expression.However, micro-arc CaP coatings prepared at an MAO voltage of 200–250 V did not statistically affect the expression of these T cell differentiation genes (*GFI1*, *U2AF1L4*, and *HNRNPLL*) (Table 2). 

It is possible that CaP-coated Ti samples only enhanced preliminary stimulation of 14-day proliferative tumor cells with anti-CD2/CD3/CD28 antibodies.

## 4. Discussion

The complex, hierarchical (Figure 1), amorphous–crystalline (Figure 2 and Figure 3) structure of CaP coatings are associated with features of their synthesis using the MAO method. MAO is a step-by-step process whereby coatings are formed layer by layer [43,53]. Such layer-by-layer deposition, accompanied by a change in current (in our case) or voltage over time, can lead to the formation of a hierarchy of microstructure, morphology, phase composition, and elemental composition in the coatings. With an increase in MAO voltage, the intensity of micro-arc discharges increases, and the amplitude and current density increase. This leads to greater heating of the electrolyte and an increase in the deposition rate of the coatings. 

The structure of a substance synthesized from a melt has been known to depend on the cooling rate. If the cooling rate is very high, the particles of the substance (atoms, molecules) cannot form a thermodynamically stable crystalline state. In this case, an amorphous solid is formed. Otherwise, under thermally stable conditions, the particles can organize a structure with a two- or three-dimensional order and form a crystalline or semi-crystalline solid [54]. In the MAO process, a coating is formed due to the action of short-lived micro-discharges migrating over the surface of a metal substrate. During the rapid cooling of the plasma in the micro-arc discharge channel, the substance is partially crystallized in the form of compounds and partially formed in an amorphous and nanostructural state [13,16].

The XRD revealed that with an increase in MAO voltage, the amount of CaP crystalline phases (CaHPO_4_, β-Ca_2_P_2_O_7_) increased from 0 to 42 vol.% and, consequently, the amount of the amorphous phase decreased from 100 to 58 vol.% (Figure 2). These data are in a good agreement with the TEM and SEM results. The micro-sized crystallites of monetite on the surface of the coating deposited at 250–300 V were observed by the SEM (Figure 1b,c). In addition, nanocrystallites of both CaHPO_4_ and β-Ca_2_P_2_O_7_were showed by TEM (Figure 3). Thus, the MAO CaP coatings have a complex polyphase composition including amorphous, nanocrystalline, and microcrystalline states.

CaP materials in an amorphous state are thermodynamically unstable and easily transform into a crystalline state. This determines their high biological activity and the ability to stimulate biomineralization [55]. In addition, it was found that calcium orthophosphates with the nanocrystalline structure stimulate a higher proliferative activity of various types of cells since they have high surface energy and reactivity [56].

As mentioned above, MAO coatings with an amorphous–nanocrystalline structure were formed under thermodynamically nonequilibrium conditions due to unsteady plasma–chemical processes and rapid plasma cooling. In this case, there is a high probability of uncompensated bonds and structural defects which determine the presence of a charge.

Studies of the coating EP revealed its high negative values in the range of −456 to −535 mV. This can be the result of the anodic polarization of the Ti sample. The negative ions contained in the electrolyte were most preferably attracted to the positive anode (sample) and deposited in the coating. This is in good agreement with our previous results of the infrared spectroscopy illustrated the intensive absorbance bands from the phosphate and hydroxyl bonds in the CaP coatings [43]. In addition, previous studies on the coating wettability showed that the polar component (responsible for strong polar chemical bonds in the coatings, e.g., phosphate and hydroxyl bonds) prevailed significantly over the dispersive component of the free surface energy [44].

**Figure 5:**
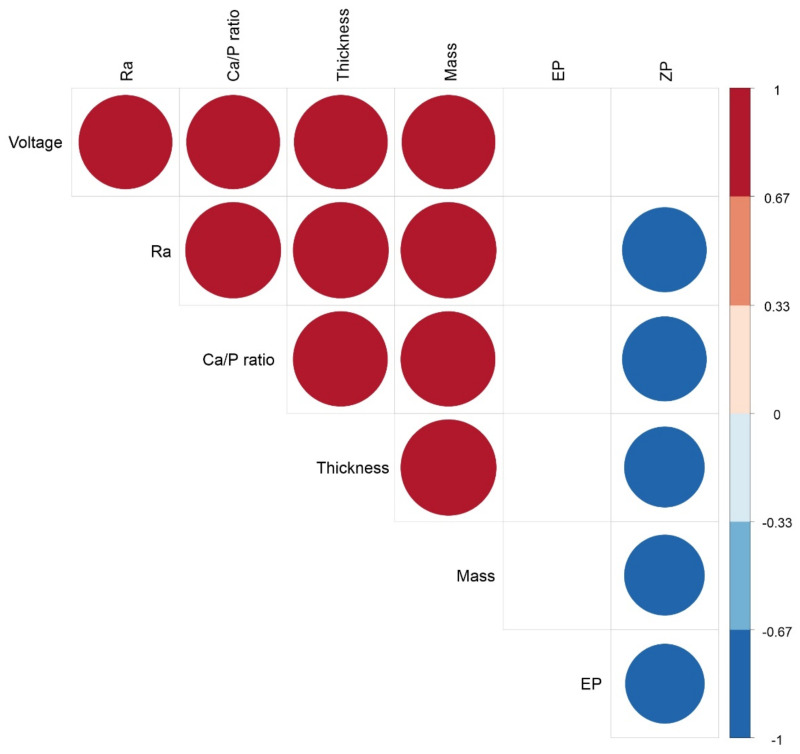
Strong relationships (*r_S_* > 0.75; *n* = 12–30) between the technological and physicochemical properties of micro-arc CaP-coated Ti substrates. Positive and negative Spearman’s rank correlations are shown by red and blue circles, respectively.

Correlation analysis showed strong (*r_S_* > 0.8; *p* < 10^−6^; *n* = 30) direct relationships (Figure 5) between technological (MAO voltage values), structural (*R_a_*, mass, and thickness), and elemental (Ca/P ratio) features of micro-arc CaP coatings on Ti plates. However, structural and elemental indices did not closely influence the EP magnitudes (*r_S_* < 0.4; *p* > 0.05; *n* = 24) of negatively charged CaP coatings. The conclusion that the EP does not strongly depend on the structural and elemental properties of micro-arc CaP-coated Ti samples was formulated and partially supported by our previous results [47]. Conversely, the ZP magnitudes of the negatively charged CaP coatings were related indirectly (*r_S_* > −0.76; *p* < 0.004; *n* = 12) to the studied physicochemical indices (Figure 5) as well as the values of MAO voltage (*r_S_* = −0.71; *p* < 0.01; *n* = 12).

Thus, the applied MAO voltage in the range of 200–300 V allowed regulation, to a certain extent, of the physico-chemical and electrical properties of CaP-coated Ti substrates. This may be a technological mechanism triggering the behavior of cells contacted with micro-arc CaP coating.

It is well known that biomaterials acquire a surface electrical charge in liquids by different processes such as ionization, ion adsorption, or ion dissolution [57]. Therefore, in biological fluids, the charge state of biomaterials can change considerably. Mesenchymal stem cells (MSCs) could be able to be recruited by an electric micro-environment through galvanotaxis to the defect area and take part in the bone healing [58]. Zhang et al. [59] demonstrated that a surface EP of approximately −77 mV generated by polarized nanocomposite PVDF-based membranes had a stimulating effect on in vitro MSC motility and osteogenic differentiation as well as in vivo rapid bone regeneration leading to complete, mature bone-structure formation. For reference, values of resting membrane potential vary in healthy and cancer cells in the physiological range of −5 to −150 mV [60,61]. 

The negative sign of the EP and its high magnitude on micro-arc CaP-coated Ti substrates have been recently proposed to be sensitive factors that can trigger the micro- and nanoscale osteoblastic differentiation and maturation of human MSCs [47] and hyperactivation-dependent death of CD4^+^ Jurkat T cells [39]. 

The first question of our study was to investigate whether micro-arc CaP coatings maintain their negative surface charge at high EP (−400–500 mV) in water, similar to uncoated Ti specimens [62]. Usually, there is a reduction in the EP in electrolytes conditioned by the electrokinetic interaction at the artificial surface and solution interface, forming a double electric layer [63]. Indeed, our results showed an 8–13-fold decrease (compared with the EP magnitudes) in the ZP values. At the same time, the CaP-coated samples prepared at the different MAO voltages were immersed into KCl with NaOH solution at pH = 7 (Figure 4a,b). However, full neutralization of negative surface charge was not observed at pH values varying from 5.4 to 8 units (Figure 4c).

The prepared micro-arc CaP coatings contained Ca-deficient amorphous and crystalline CaHPO_4_ and β-Ca_2_P_2_O_7_ phases (Figure 2 and Figure 3) and hadlow Ca/P atomic ratios in the range of 0.3–0.6 (Figure 4b). Calcium phosphates with low Ca/P atomic ratios and an excess amount of H_2_PO_4_ groups were negatively charged around pH 7.40 [64,65]. For example, the negative sign of the ZP on monetite particles with grain sizes of 1–20 µm immersed in 0.05 M sodium phosphate solution achieved a value of −45 mV [66]. Of note, this value almost aligns with those of the tested CaP coatings (Figure 4c).

We recently demonstrated in vitro dissolution of the used micro-arc CaP coating [44] accompanied by both ion (Ca^2+^ and PO_4_^3−^) and CaP microcrystallite (possibly, monetite) release [67]. The ever-increasing popularity of monetite as a biomaterial for biomedical implants is explained by its better balance between resorption and new bone formation as compared to other CaP phases. CaHPO_4_ particles are soluble in bodily fluids and promote additional ion elution, nucleation, and growth of bone apatite during the biomineralization. Moreover, its biological (in vitro and in vivo) properties are discussed [68].

The ZP magnitude and sign of Ca-deficient CaP coatings are related to their EP as well as ion exchange between the hydrated layer around the surface and net precipitation of new material. A reverse of the negative charge of the ZP of CaP material occurs if the immersion time exceeds 2 days at pH 7.0. This depends on proton, hydroxyl, calcium, phosphate ion extraction, and changes in the electrical double layer surrounding a CaP surface solution [64]. Similarly, negatively charged micro-arc CaP surfaces with a high *R_a_* index, Ca/P atomic ratio (Figure 4a,b), mass, and thickness of the coating deposited at 300 V showed statistically lower ZP magnitudes (Figure 4c) than those received at 200–250 V. Due to the more complex nature of ZP formation, the Ti substrates coated by CaPs at an MAO voltage of 300 V were excluded from further experiments with cells.

Moreover, the high applied voltages (>250 V) strongly decreased (to below 12 MPa) the adhesion strength of the micro-arc CaP coatings to the Ti-based substrate [43]. The coating adhesion strength for medical devices must exceed the value of 15 MPa according to ISO 13779-2:2018 Implants for Surgery—Hydroxyapatite—Part 2: Thermally Sprayed Coatings of Hydroxyapatite. 

The second item of our study was to estimate the in vitro effect of CaP-coated samples on in vitro behavior of tumor-derived Jurkat T cells. We have previously described an in vitro inhibiting action of micro-arc CaP-coated Ti samples on the resting (naïve) Jurkat T cells and proposed the negative ZP and Ca^2^^+^ effectors of CaP roughness [39]. Values of ZP less than −30 mV are considered strongly anionic and can influence cells [69]. Therefore, the ZP of CaP ceramics is related to electrokinetic potentials known to cause a substantial effect on the cell activities when applied exogenously [64].

In the current research, Jurkat T cells were activated by beads coated with a mitogenic mixture of anti-CD2/CD3/CD28 antibodies before 14-day in vitro contact with CaP-coated specimens. This experimental model mimics a situation when leukemic immune cells invade the bone marrow and other tissues and are exposed to the stimulating signals produced by APCs. They reach the bone and/or implant surfaces in oncological patients. In vitro simulation of cell–cell interaction of tumor T lymphoblasts with APCs was accompanied by enhanced survival of CD4^+^, CD4^+^CD71^+^, and CD4^+^CD95^+^ subsets of CD45^+^CD3^+^ Jurkat T cells (Table S2) as well as a modified spectrum of the produced biomolecules with a statistically significant increase in VEGF and MIP-1β (CCL4 chemokine) concentrations (Table S3) that promotes cancer cell proliferation and metastasis [70,71]. CD95 activation can trigger a migration of tumoral cells [42] and invasion of Jurkat line cells [72], displaying resistance to the CD95-mediated apoptotic signal. CD71 (transferrin receptor) recruitment was observed in response to mitogenic anti-CD3 and CD28 antibodies, and CD71 involvement in forming the immunological synapses in Jurkat T cell-APC contacts is proposed as an activating stimulus [73]. Moreover, IL-8 secretion decreased approximately six-fold (Table S3). IL-8 is an apoptosis-inducing factor for leukemic cell lines (Jurkat, K562, HL-60, etc.) [74]; its close (99%) relationship with Jurkat T cell necrosis in vitro is predicted in the case of CaP-based micro-arc coatings [75].

hTERT (human telomerase reverse transcriptase) possesses reverse transcription activity and synthesizes the telomeric repeats on the human telomerase RNA template, comprising the RNA template for the synthesis of DNA telomeres [52,76]. Telomerase activity maintains the lifespan and immortalizes about 90% of malignant cells [77], including Jurkat T cells [52]. 

Tumor-derived Jurkat T cells preactivated with anti-CD2/CD3/CD28 antibodies strongly responded to the micro-arc CaP coating and showed a 4.5-fold upregulation of hTERT gene expression after 14-day culture (Table 2). In turn, this underlined the in vitro survival of CD4^+^ subsets of activated tumor-derived Jurkat T cells (Table S2) and their proinflammatory secretion (Table S3) during 14-day contact with rough CaP coating bearing a negative sign of ZP (Figure 4b,c). This was a little unexpected since we previously described a suppressing action of micro-arc rough CaP-coated and uncoated Ti substrates on the in vitro lifespan of naïve Jurkat T cells [39,49].

Hence, the third critical question of our study was the possible mechanism underlying the obtained effect. We showed close correlations between CaP coating features (*R_a_* index, thickness, mass, and the ZP value) (Figure 5) and molecular (gene and secretory) activities of Jurkat T cells (see above). Understandably, there are at least three mediators, namely the ZP, Ca flux, and micro- and nanoparticles released from micro-arc CaP coating which can promote the in vitro survival of activated Jurkat T cells. However, the ZP value mainly impacts tumor cells (via sedimentation and adherence) in direct contact with CaP surfaces [49]; Ca^2+^ and CaP crystallites released during CaP biodegradation [67] may also influence the suspension fraction of tumor-derived cells.

Extracellular Ca^2^^+^elevation has been shown to briefly provoke plasma membrane depolarization (reduction) [78] and intracellular Ca^2^^+^augmentation [79]. Stimulation of the T cell receptor (TCR) with CD3-specific antibodies led to increased intracellular free Ca^2+^ concentration in activated Jurkat cells. As a result, TCR functioning as a Ca^2+^-mobilizing transmembrane receptor during T cell activation was concluded [80]. Simultaneously, an increased cytoplasmic Ca^2+^ concentration neutralized negatively charged membranes in the T cell activation zone of the TCR-CD3 complex to form an immunological synapse and initiate or amplify TCR signaling microclusters in Jurkat cells [40]. TCR and CD3 ligands and Ca^2+^ ionophores behaved as interchangeable activating stimuli for Jurkat cells [40,81]. Thereby, CD2CD3CD28-specific antibodies and extracellular calcium released from micro-arc CaP coatings served as synergistic factors to stimulate Jurkat T cell behavior in the presence of test samples.

Micro-arc CaP-coated Ti samples are moderately soluble (approximately 0.25 mM per week) [67]. Additionally, extracellular Ca^2+^ levels at different pH values may regulate the ZP negative charge due to increasing Ca/P atomic ratios of CaP coatings prepared at 200–250 V (Figure 1b,c) and ion redeposition (precipitation) on CaP surfaces [39]. This may reverse the ZP sign during 14-day immersion in cell suspension as described by Ducheyne et al. [64] for fluid solutions. Therefore, other physical–chemical irritants of micro-arc CaP coatings (monetite crystallites, ZP) should be considered.

Details of the exact pathways underlying the synergistic enhancing effect of negatively charged CaP surfaces (coatings and CaHPO_4_ particles) with anti-CD2CD3CD28 antibodies on in vitro survival and *hTERT* expression in Jurkat T cells remain elusive. Jurkat cells bear negative ZP on the cell surface caused by negatively charged membrane glycoproteins [82] with the carboxyl groups of sialic acids [83]. Subsequently, trace amounts (0.01%) of Jurkat E6.1 leukemic lymphoblasts uptake PEGylated polyglutamic acid capsules with a mean size of 116 nm and negative zeta potential of −29 mV [84]. Moreover, Rahman et al. [85] reported the in vitro cytotoxicity rather than the activating action of negatively charged nanostructured lipid carriers on Jurkat cells after 72 h of treatment. Microbeads coated with anti-CD3 and anti-CD28 antibodies causeless negatively charged membranes in the Jurkat T cell stimulation zone (the immunological synapse) than in regions of the membrane where the TCR is not activated [40]. Micro-arc CaP-coated Ti samples and monetite crystallites possessed negative ZP values (Figure 4b) [66]. Therefore, the impact of their direct activation on the immunological synapse of excited membranes of Jurkat T cells could be excluded if there is not charge inversion caused by Ca^2+^ redeposition on artificial surfaces from the solution.

According to obtained results and discussion, the ZP and transmembrane potential (TMP) of Jurkat T cells may be targeted by the negative charge of CaP coatings and monetite particles. Experimental findings of real connections are practically absent. In part, the ZP is closely related to the TMP of leukocytes cultured in vitro, and the ZP value is affected by the fixed charges outside the cell membrane [86]. However, the authors emphasized that there was no general association between the changes in ZP and TMP. Indeed, Yao et al. [87] proposed that a positive charge on nanoparticle surfaces induces membrane depolarization, thus theoretically providing downstream intracellular events that activate macrophages. On the other hand, ZnO nanoparticles with a negative ZP (19 mV) exhibit interaction with cell membrane proteins via hydrogen bond interaction with amino acid residues followed by internalization and membrane depolarization [88].

Electric fields of magnitudes similar to those measured in vivo have been shown to direct polarity in living cells and tissues. However, how cells sense EF signals and reorganize the cytoskeleton, intracellular pH, and polarity machinery is poorly documented. Electric fields may influence ion transport and/or membrane potential locally around the cell [89]. The negatively charged ZP of the CaP surfaces is comparable to the TMP of Jurkat cells and could perhaps enhance the membrane fluctuations of preliminary activated tumor-derived cells, resulting in an intracellular [Ca^2^^+^] increase via different membrane potential-regulating ion channels [90] and the change in the cation distribution in the extracellular space [91]. Changes in the membrane potential, in turn, could indirectly influence downstream cytoplasmic factors and gene transcription [92].

**Figure 6:**
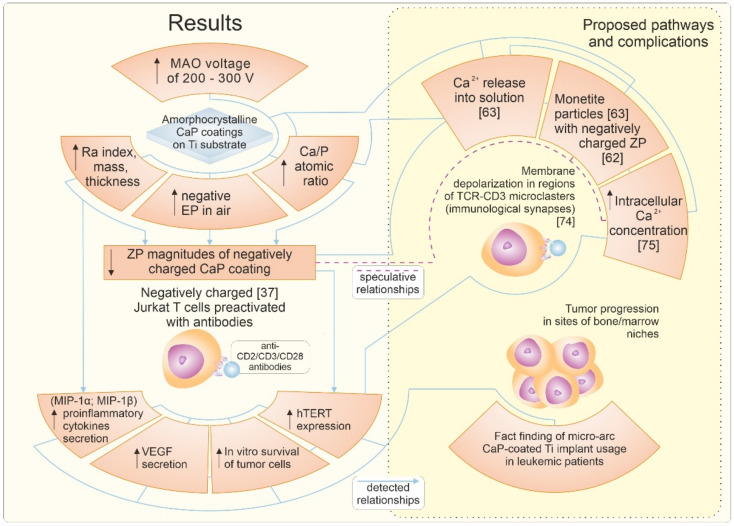
Results and possible Jurkat T cell behavior pathways in conditions of 14-day in vitro costimulation with anti-CD2/CD3/CD28 antibodies and rough micro-arc CaP-coated Ti substrates.

In summary, the primary data and possible pathways are presented in Figure 6.

Of interest are the proposed implications of these findings. Bone marrow (BM) and the endosteal surface of bone represent specific micro-environment territories (niches) that are poised to maintain hematopoietic stem cells (HSCs), including lymphoid progenitors [93]. Endosteal (osteoblastic) microterritories encompass the bone matrix, the inner surface that regulates the interconnections of MSCs, bone cells, and HSCs in the hierarchically subdivided quiescent and activated niches [94]. 

In turn, healthy niches can provide an attractive milieu for leukemic cell colonization, given the ability to provide signals that influence tumor cell survival [88] that are still niche dependent. It is known that the niches maintain the survival of leukemic stem cells in a quiescent state [95]. Furthermore, tumor lymphoid cells can rearrange a micro-environment towards their needs [96,97,98]. 

We propose that rough micro-arc CaP surfaces contain specialized artificial microterritories (niches) that simulate the bone milieus for MSC differentiation into osteoblasts and HSC regulation [99]. Based on our results, functional profiles (naïve or activated) of leukemic Jurkat T lymphoblast-like cells allow them to use the cues produced by the niche differently. The contact between APCs and tumor cells metastasizing the bone and marrow tissues may dramatically replace quiescent signals by activating stimuli produced by niches and, thus, promote further tumor growth progression.

## 5. Conclusions

Despite the limitations of the in vitro study, we found that rough CaP micro-arc coatings with an amorphocrystalline structure generated negative EP values in air and negative ZP in water solution. MAO method may be used as a technological tool to trigger the behavior of cells through contact with micro-arc CaP coatings. There is an opinion that negative values of EP and ZP should be carefully considered in biomaterial design, and this concept may provide an innovative and well-suited strategy for regenerative bone surgery [59]. Based on our data, a decrease in the ZP magnitudes of CaP coatings deposited at 200–250 V was strongly associated with elevated *hTERT* expression in tumor-derived Jurkat T cells preliminarily activated with anti-CD2/CD3/CD28 antibodies and then contacted in vitro with CaP-coated samples for 14 days. Thereafter, in vitro survival of CD4^+^ subsets was enhanced, with proinflammatory cytokine secretion of activated Jurkat T cells. In this regard, micro-arc CaP coatings should be adequately tested to determine their suitability for use in implants meant for patients with chronic lymphoid malignancies. Future studies are necessary to confirm whether these results can be applied to actual clinical situations in the orthopedic and dental fields.

## Figures and Tables

**Table 1:** Sequences of oligonucleotide primers used in the experiment.

*hTERT* (*human telomerase reverse transcriptase*) *hTERT*_for5′-TGACACCTCACCTCACCCAC-3′ *hTERT*_rev5′-CACTGTCTTCCGCAAGTTCAC-3′
*U2AF1L4* (*U2 small nuclear RNA auxiliary factor 1 like 4*) *U2AF1L4*_for 5′-CTTCACAACAAGCCGACATTC-3′ *U2AF1L4*_rev 5′-CAAGGTTGTCGCACACATTC-3′
*GFI1* (*growth factor independent 1 transcriptional repressor*) *GFI1*_for5′-TGGAGCAGCACAAAGCC-3′ *GFI1*_rev 5′-GACAGTGTGGATGACCTCTTG-3′
*HNRNPLL* (*heterogeneous nuclear ribonucleoprotein L*) *HNRNPLL*_for5′-CTCTCAATTCAGAATCCGCTTTATC-3′ *HNRNPLL*_rev 5′- CCATTGCTTGTATCCCATTTCTC-3′
*RPLPO* (*large ribosomal protein*) *RPLPO*_for 5′-GGCGACCTGGAAGTCCAACT-3′ *RPLPO*_rev 5′-CCATCAGCACCACAGCCTTC-3′
*hTERT*_probe FAM-5′-ACCCTGGTCCGAGGTGTCCCTGAG-3′-BHQ-1 *U2AF1L4*_probe FAM-5′-CCAGGAGGTGTTCACAGAACTGCA-3′~BHQ-1 *GFI1*_probe FAM-5′-CGCAGGAACGGAGCTTTGACTGTA-3′~BHQ-1 *HNRPLL*_probe FAM-5′-TATGCAACCCTGTTGGCAAAGTGC-3′~BHQ-1 *RPLPO*_ probe Bgl635-5′-ATCTGCTGCATCTGCTTGGAGCCCA-3′-BHQ-2

**Table 2:** Relative gene expression levels (fold) in Jurkat T cells preactivated with anti-CD2/CD3/CD28 antibodies and collected from plastic after 14 days of in vitro coculture with the micro-arc CaP-coated titanium substrates, Me (Q1; Q3).

Parameters of Bilateral CaP Coating on a Titanium Substrate, *n* = 3 ^a^			Relative Expression of Genes			
Ra, µm	Thickness, µm	Weight, mg	*hTERT*	*U2AF1L4*	*GFI1*	*HNRNPLL*
3.1 (2.5; 4.7)	53.0 (39.5; 70.5)	14.5 (10.6; 19.1)	4.49 ^b^ (1.80; 7.01)	−1.0 (−1.36; 4.11)	1.34 (−2.69; 1.57)	65.50 (−50.70; 168.39)

^a^*n*: the number of test samples; ^b^*p* < 0.05 vs. control according to the Mann–Whitney U test; (−) sign means inhibition of relative gene expression in comparison with a control cell culture (activated Jurkat T cells without CaP-coated substrates); expression of each gene was measured in triplicate.

## Data Availability

The data presented in this study are available within the article and Supplementary Materials.

## Supplementary Materials

The following are available online at https://www.mdpi.com/article/10.3390/ma14133693/s1. Table S1: Cytokine concentrations (pg/mL) in the supernatants of Jurkat T cells cultured in the different mediums for 14 days; Me (Q1; Q3); Table S2: Viability and immunophenotype of CD45^+^CD3^+^ Jurkat T cells preactivated with anti-CD2/CD3/CD28 antibodies and collected from plastic after 14 days of in vitro coculture with the CaP-coated Ti samples; Me (Q_1_; Q_3_); Table S3: Cytokine concentrations (pg/mL) in the supernatants of Jurkat T cells preactivated with anti-CD2/CD3/CD28 antibodies and cultured for 14 days in the presence of the CaP-coated Ti samples; Me (Q1; Q3).
