# Supplementary material for: Amorphous–Crystalline Calcium Phosphate Coating Promotes In Vitro Growth of Tumor-Derived Jurkat T Cells Activated by Anti-CD2/CD3/CD28 Antibodies
Source: Materials (Basel). 2021 Jul 1;14(13):3693. doi: 10.3390/ma14133693 (PMC8269898; doi:10.3390/ma14133693)
Supplement: Supplementary file 1 [file materials-14-03693-s001.zip › materials-1248469-supplementary.pdf]

Supplementary Material

Amorphous–Crystalline Calcium Phosphate Coating Promotes In Vitro Growth of Tumor-derived Jurkat T Cells Activated by anti-CD2/CD3/CD28 Antibodies

Yurii P. Sharkeev [1,2], Ekaterina G. Komarova [1], Valentina V. Chebodaeva [1], Mariya B. Sedelnikova [1], Aleksandr M. Zakharenko [3], Kirill S. Golokhvast [3], Larisa S. Litvinova [4], Olga G. Khaziakhmatova [4], Vladimir V. Malashchenko [4], Kristina A. Yurova [4], Natalia D. Gazatova [4], Ivan G. Kozlov [5], Marina Y. Khlusova [6], Konstantin V. Zaitsev [7], and Igor A. Khlusov [4,8,9]

Citation: Sharkeev, Y.P.; Komarova, E.G.; Chebodaeva, V.V.; Sedelnikova, M.B.; Zakharenko, A.M.; Golokhvast, K.S.; Litvinova, L.S.; Khaziakhmatova, O.G.; Malashchenko, V.V.; Yurova, K.A.; et al. Amorphous–Crystalline Calcium Phosphate Coating Promotes In Vitro Growth of Tumor-Derived Jurkat T Cells Activated by Anti-CD2/CD3/CD28 Antibodies. *Materials* **2021**, *14*, 3693. https://doi.org/10.3390/ma14133693

AcademicEditor: **Frédéric Velard**

Received: 20 May 2021
Accepted: 25 June 2021
Published: 1 July 2021

Publisher's Note: MDPI stays neutral with regard to jurisdictional claims in published maps and institutional affiliations.

Copyright: © 2021 by the authors. Licensee MDPI, Basel, Switzerland. This article is an open access article distributed under the terms and conditions of the Creative Commons Attribution (CC BY) license (http://creativecommons.org/licenses/by/4.0/).

[1] Laboratory of Physics of Nanostructured Biocomposites, Institute of Strength Physics and Materials Science, Siberian Branch of Russian Academy of Sciences, Tomsk 634055, Russia; sharkeev@ispms.tsc.ru (Y.P.S.); katerina@ispms.ru (E.G.K.); vtina5@mail.ru (V.V.C.); smasha5@yandex.ru (M.B.S.)

[2] Research School of High-Energy Physics, National Research Tomsk Polytechnic University, Tomsk 634050, Russia

[3] School of Engineering, Far Eastern Federal University, Vladivostok 690090, Russia; rarf@yandex.ru (A.M.Z.); golokhvast.ks@dvfu.ru (K.S.G.)

[4] Center for Immunology and Cellular Biotechnology, Immanuel Kant Baltic Federal University, Kaliningrad 236029, Russia; hazik36@mail.ru (O.G.K.); vlmalashchenko@kantiana.ru (V.V.M.); kristina_kofanova@mail.ru (K.A.Y.); n_gazatova@mail.ru (N.D.G.)

[5] Department of Organization and Management in the Sphere of Circulation of Medicines, Institute of Postgraduate Education , I.M. Sechenov Federal State Autonomous Educational University of Higher Education—First Moscow State Medical University of the Ministry of Health of the Russian Federation (Sechenov University), Moscow 119991, Russia; immunopharmacology@yandex.ru

[6] Department of Pathophysiology, Siberian State Medical University, Tomsk 634050, Russia; marikhl@mail.ru

[7] Siberian Federal Scientific and Clinical Center of the Federal Medical-Biological Agency, Seversk 636070, Russia; zaitsev-kv@mail.ru

[8] Research School of Chemistry and Applied Biomedical Sciences, National Research Tomsk Polytechnic University, Tomsk 634050, Russia

[9] Department of Morphology and General Pathology, Siberian State Medical University, Tomsk 634050, Russia

* Correspondence: larisalitvinova@yandex.ru (L.S.L.); khlusov63@mail.ru (I.A.K.); Tel.: +7-4012-595-595 (ext. 6631) (L.S.L.); +8-3822-901-101 (ext. 1823) (I.A.K.)

Table S1. Cytokine concentrations (pg/mL) in the supernatants of Jurkat T cells cultured in the different mediums for 14 days; Me (Q1; Q3).

IL-1β	IL-1Ra	IL-2	IL-4	IL-5	IL-6	IL-7	IL-9	IL-10	IL-12 (p70)	IL-13	IL-15	IL-17	TNFα	IFNγ
RPMI-1640 medium, n = 4														
0.13 (0.07; 0.14)	9.29 (3.10; 13.31)	0 (0; 0.72)	0.49 (0.31; 0,67)	0.19 (0; 0.46)	1.34 (0,59; 2.11)	1.62 (1.30; 1.92)	0.50 (0.24; 0.82)	0.24 (0.04; 0.63)	0.76 (0.33; 6,09)	0.29 (0; 0.64)	0 (0; 0)	0.34 (0; 1.25)	1.64 (0.78; 1.83)	23.31 (7.01; 40.75)
αMEM medium, n = 4														
0.29* (0.27; 0.39)	26.09* (12.07; 37.71)	0.49 (0.24; 0.88)	0.77 (0.58; 0,93)	0 (0; 0)	25.50* (23.22; 27.18)	2.26 (1.61; 2.77)	4.36* (3.88; 5.30)	22.78* (21.52; 27.15)	107.54* (99.57; 117.54)	2.26* (1.40; 2.89)	0 (0; 0)	0.28 (0.12; 7.43)	10.01* (8.56; 12.11)	45.83 (16.84; 66.94)

Table S1. In continuous

bFGF	VEGF	PDGF-BB	G-CSF	GM-CSF	IL-8 (CXCL8)	Eotaxin (CCL11)	IP-10 (CXCL10)	MCP-1 (CCL2)	MIP-1α (CCL3)	MIP-1β (CCL4)	RANTES (CCL5)
RPMI-1640 medium, *n* = 4											
13.62 (10.68; 15.05)	2.08 (1.82; 2.62)	1.88 (1.50; 2.02)	1.32 (0.64; 2.07)	29.73 (23.50; 35.41)	0.64 (0.49; 1.23)	2.22 (2.06; 2.24)	8.16 (4.98; 10.62)	3.06 (2.89; 3.82)	0.13 (0.11; 0.19)	0.94 (0.57; 1.10)	0 (0; 0.07)
αMEM medium, *n* = 4											
13.78 (11.16; 17.27)	2125* (1822; 2441)	3.29 (1.67; 4.34)	17.51* (15.35; 20.50)	57.29* (44.46; 68.22)	8.29* (7.67; 8.95)	6.52* (6.42; 6.78)	19.90* (13.92; 24.90)	10.25* (10.08; 11.55)	0.37* (0.31; 0.39)	6.48* (6.19; 6.58)	1.12* (0.85; 1.33)

Note: * P<0.05 compared with the corresponding values in RPMI-1640 medium according to the Mann-Whitn ey *U* test. Duplicate probes for each well were measured.

Table S2. Viability and immunophenotype of CD45⁺CD3⁺ Jurkat T cells preactivated with anti-CD2/CD3/CD28 antibodies and collected from plastic after 14 days of in vitro coculture with the CaP-coated Ti samples; Me (Q_1 ; Q_3).

R_a, μm	**Live or Dead Cells, %**			**Cells Expressing Specific Membrane Markers, %**						
	**Viable Cells**	**Apoptosis**	**Necrosis**	**CD4**	**CD8**	**CD4CD71**	**CD4CD25**	**CD4CD95**	**CD4CD45RA**	**CD4CD45R0**
1) Tumor cells on plastic surface (control), n = 3										
	90.92	2.88	6.29	62.16	2.77	70.38	27.88	39.03	99.36	39.71
-	(90.18; 91.98)	(2.79; 2.99)	(5.14; 6.83)	(59.78; 62.80)	(2.51; 3.77)	(69.75; 73.12)	(26.66; 29.04)	(38.28; 45.24)	(99.29; 99.42)	(35.36; 41.43)
2) Preliminary activated tumor cells on plastic surface, n = 3										
-	92.89	2.25	4.62	84.80	2.02	77.90	31.58	71.35	98.48	33.56
	(92.49; 93.78)	(2.06; 2.49)	(4.16; 5.26)	(81.30; 84.95)	(0.51; 3.46)	(74.56; 79.15)	(28.37; 31.98)	(68.49; 78.68)	(98.30; 98.85)	(31.75; 39.58)
	$P_1<0.05$	$P_1<0.05$		$P_1<0.05$		$P_1<0.05$		$P_1<0.05$	$P_1<0.05$	
3) Preliminary activated tumor cells in contact with the CaP-coated Ti samples, n = 3										
3.20	93.90	2.08	3.94	83.51	1.52	77.70	30.80	70.64	98.41	32.46
(2.14; 3.40)	(93.84; 95.15)	(1.78; 2.16)	(2.77; 4.38)	(82.88; 85.05)	(1.38; 1.78)	(76.48; 78.11)	(30.14; 32.63)	(68.37; 72.82)	(98.40; 98.60)	(31.44; 35.36)
	$P_1<0.05$	$P_1<0.05$	$P_1<0.05$	$P_1<0.05$	$P_1<0.05$	$P_1<0.05$	$P_1<0.05$	$P_1<0.05$	$P_1<0.05$	
	$P_2<0.05$									

Note: P_n, significant difference (<0.05) compared with the corresponding group number according to the Mann-Whitney test. Duplicate probes for each well were measured.

Table S3. Cytokine concentrations (pg/mL) in the supernatants of Jurkat T cells preactivated with anti-CD2/CD3/CD28 antibodies and cultured for 14 days in the presence of the CaP-coated Ti samples; Me (Q1; Q3).

**Bilateral CaP Coating**			**Inflammatory Interleukins and Cytokines**														
***R_a*, μm**	**Thickness, μm**	**Mass, mg**	**IL-1β**	**IL-1Ra**	**IL-2**	**IL-4**	**IL-5**	**IL-6**	**IL-7**	**IL-9**	**IL-10**	**IL-12 (p70)**	**IL-13**	**IL-15**	**IL-17**	**TNFα**	**IFNγ**
1) Tumor cells on plastic surface, *n* = 5																	
0	0	0	0.37 (0.36; 0.37)	40.08 (36.56; 45.06)	0.92 (0.68; 1.37)	1.0 (0.67; 1.09)	0 (0; 0.54)	487 (479; 491)	1.94 (1.81; 3.03)	3.88 (3.31; 4.32)	19.58 (19.29; 20.39)	88.52 (88.45; 89.48)	2.44 (1.94; 2.57)	0 (0; 0)	1.20 (0.43; 2.52)	9.99 (9.03; 12.81)	51.16 (29.56; 53.73)
2) Preliminary activated tumor cells on plastic surface, *n* = 4																	
0	0	0	0.29 (0.27; 0.39)	26.09 (12.07; 37.71)	0.49 (0.24; 0.88)	0.77 (0.58; 0.93)	0 (0; 0)	25.50 (23.32; 27.18) $P_1<0.001$	2.26 (1.61; 2.77)	4.36 (3.88; 5.30)	22.78 (21.52; 27.15)	108 (100; 118) $P_1<0.02$	2.26 (1.40; 2.89)	0 (0; 0)	0.28 (0.12; 7.43)	10.01 (8.56; 12.11)	45.83 (16.84; 66.94)
3) Preliminary activated tumor cells in contact with the CaP-coated Ti samples, *n* = 12																	
3.1 (2.5; 4.7)	53.0 (39.5; 70.5)	14.5 (10.6; 19.1)	0.30 (0.22; 0.63)	25.69 (13.17; 36.84)	1.55 (1.19; 2.37)	0.92 (0.74; 1.09)	0 (0; 0)	1.08 (0.37; 154) $P_1<0.001$	2.16 (1.73; 3.31)	3.88 (3.54; 5.78)	27.31 (22.59; 31.81) $P_1<0.04$	116 (103; 148) $P_1<0.02$	2.28 (2.09; 2.65)	0 (0; 0)	1.53 (0.63; 3.10)	11.07 (9.46; 13.97)	40.45 (16.60; 55.65)

Table S3. In continuous

Angiogenic Molecules			Hematopoietic Growth Factors		Chemokines						
bFGF	VEGF	PDGF-BB	G-CSF	GM-CSF	IL-8 (CXCL8)	Eotaxin (CCL11)	IP-10 (CXCL10)	MCP-1 (CCL2)	MIP-1α (CCL3)	MIP-1β (CCL4)	RANTES (CCL5)
1) Tumor cells on plastic surface, *n* = 5											
14.48 (14.47; 17.95)	1735 (1707; 1768)	2.16 (2.14; 3.83)	43.77 (39.28; 45.67)	58.94 (55.33; 59.20)	47.69 (47.57; 47.70)	10.49 (9.97; 10.79)	21.27 (19.49; 22.53)	19.55 (18.57; 20.02)	0.29 (0.25; 0.39)	4.58 (4.58; 4.63)	1.32 (1.26; 1.52)
2) Preliminary activated tumor cells on plastic surface, *n* = 4											
13.78 (11.16; 17.27)	2125 (1822; 2441) $P_1<0.05$	3.29 (1.67; 4.34)	17.51 (15.35; 20.50) $P_1<0.001$	57.29 (44.46; 68.22)	8.29 (7.67; 8.95) $P_1<0.001$	6.52 (6.42; 6.78) $P_1<0.004$	19.90 (13.92; 24.90)	10.25 (10.08; 11.55) $P_1<0.001$	0.37 (0.31; 0.39)	6.48 (6.19; 6.58) $P_1<0.006$	1.12 (0.85; 1.33)
3) Preliminary activated tumor cells in contact with the CaP-coated Ti samples, *n* = 12											
17.01 (13.48; 20.28)	2116 (1411; 2706)	3.20 (1.96; 3.84)	14.39 (10.99; 57.07)	54.99 (46.64; 63.71)	5.36 (5.25; 6.57) $P_1<0.004$	6.47 (5.47; 8.53) $P_1<0.02$	21.20 (18.66; 24.19)	11.25 (9.38; 14.53) $P_1<0.001$	0.53 (0.45; 0.60) $P_1<0.001$ $P_2<0.002$	9.60 (8.34; 10.77) $P_1<0.001$ $P_2<0.002$	1.49 (1.08; 1.77)

Note: P_n, significant difference (<0.05) compared with the corresponding group number according to the Mann-Whitney *U* test. Duplicate probes for each well were measured.
